# Weight maintenance and gain were significantly associated with lower risk of all-cause and cancer-related mortality in Korean adults who were newly diagnosed with cancer based on the Korean NHIS-HEALS cohort

**DOI:** 10.1097/MD.0000000000036184

**Published:** 2023-11-24

**Authors:** Yong-June Kim, Seung Park, Won Tae Kim, Yoon-Jong Bae, Yonghwan Kim, Hee-Taik Kang

**Affiliations:** a Department of Urology, Chungbuk National University Hospital, Cheongju, Chungbuk, South Korea; b Department of Urology, Chungbuk National University College of Medicine, Cheongju, Chungbuk, South Korea; c Department of Biomedical Engineering, Chungbuk National University Hospital, Cheongju, Chungbuk, South Korea; d Department of Information & Statistics, Chungbuk National University College of Science, Cheongju, Chungbuk, South Korea; e Department of Family Medicine, Chungbuk National University Hospital, Cheongju, Chungbuk, South Korea; f Department of Family Medicine, Severance Hospital, Yonsei University College of Medicine, Seoul, South Korea.

**Keywords:** body weight changes, healthy lifestyle, malignant, mortality, neoplasms, weight loss

## Abstract

The burden of malignant neoplasms is increasing worldwide. Healthy lifestyles such as maintaining a healthy body weight are important to improve survival rate in cancer patients. This study was aimed to test the hypothesis that weight change affects mortality in patients newly diagnosed with cancer. This study was retrospectively designed based on the National Health Insurance Service-National Health Screening Cohort. A total of 1856 subjects aged at least 40 years who received a national health checkup within 6 months before cancer diagnosis was included. Study subjects were classified into 3 categories based on weight change before and after cancer diagnosis: weight loss, maintenance, and gain. Cox proportional hazards regression models were adopted to examine the association between weight change and mortality after adjusting for confounders. Compared to those experiencing weight loss, the adjusted hazards ratios (HRs) (95% confidence intervals [CIs]) for those experiencing weight maintenance were 0.327 (0.189−0.568) for all-cause mortality and 0.431 (0.215−0.867) for cancer-related mortality. The adjusted HRs (95% CIs) for those experiencing weight gain were 0.149 (0.044−0.505) for all-cause mortality and 0.289 (0.080−1.045) for cancer-related mortality. After stratifying according to baseline body mass index (BMI), weight maintenance and gain were negatively associated with all-cause mortality (0.286 [0.138−0.592] for weight maintenance and 0.119 [0.027−0.533] for weight gain) among those with a BMI < 25 kg/m^2^. Weight maintenance and gain reduced the risk of all-cause mortality in patients newly diagnosed with any cancer. In addition, weight maintenance was significantly related to cancer-related mortality.

## 1. Introduction

Statistics Korea reported that malignant neoplasms are the most common cause of death in South Korea.^[1]^ In 2020, the mortality rate (160.1 per 100,000 persons) due to malignant neoplasms was higher than the sum (105.5 per 100,000 persons) of mortality rates for cardiovascular diseases and cerebrovascular diseases in South Korea (hereafter, Korea).^[1]^ The burden of malignant neoplasms has increased globally, including in Korea.^[2–4]^ Although the best options to ameliorate the burden of malignant neoplasms are to prevent or diagnose cancer at an early stage, a healthy lifestyle is important for those diagnosed with cancer. For early detection of cancer, the Korean government provides target population National Cancer Screening Programs for 5 types of cancers (stomach, liver, colorectum, breast, and uterine cervix).^[5]^ In addition, the Korean Ministry of Health and Welfare and the National Cancer Center launched the Korean Cancer Survivorship Center Pilot Project in 2017 for long-term cancer outcomes and quality of life of cancer survivors.^[6]^ Korean Cancer Survivorship Center Pilot Project informs long-term cancer survivors on healthy lifestyles including healthy body weight maintenance, smoking cessation and alcohol consumption, and management of distress, vaccinations, social welfare services such as financial support and return to work, and health examinations for early screening for second primary cancers.

Although obesity increases the probability of various chronic diseases including cancers and cardiovascular diseases, overweight and mildly obese populations tend to live longer than populations with normal body weight.^[7]^ However, body weight loss or gain of over 5% is significantly associated with higher risk of mortality than maintaining body weight.^[8]^ In contrast, obesity or weight gain after diagnosis increased all-cause or cancer-specific mortality and recurrence among patients with breast cancer.^[9]^ Moderate weight gain reduced the risk for mortality in non-small cell lung cancer patients, and moderate weight loss was associated with a worse survival rate.^[10]^ Thus, healthy body weight is important in preventing cancer and improving health outcomes including cancer-related mortality and control of other chronic diseases.^[11]^ Several Korean studies have demonstrated that weight loss increases all-cause mortality in Koreans.^[12,13]^ In addition, many studies have investigated the association between baseline body mass index (BMI) and mortality.^[14,15]^ However, long-term outcomes that examine the association between weight change and mortality in Korean adults newly diagnosed with cancer are lacking.

The aim of this study was to test the hypothesis that weight change affects all-cause and cancer-related mortality in patients newly diagnosed with cancer based on the Korean National Health Insurance Service-Health Screening (NHIS-HEALS) cohort.

## 2. Methods

### 2.1. Study population

The Korean NHIS-HEALS cohort was comprised of 514,527 individuals who were randomly selected as a 1% sample of the 5.1 million examinees of the Korean national health examination program between 2002 and 2003. Almost all individuals were between the ages of 40 and 79 years at the end of 2002. This cohort includes information on past medical histories and lifestyles such as alcohol consumption, tobacco use, and physical activity from self-reported questionnaires, personal medical records (prescriptions and diagnostic codes) from the national health insurance claim database, death information (the date and causes of death) based on the death certificate, and anthropometric and laboratory data from the physical examination programs.

**Figure 1.**
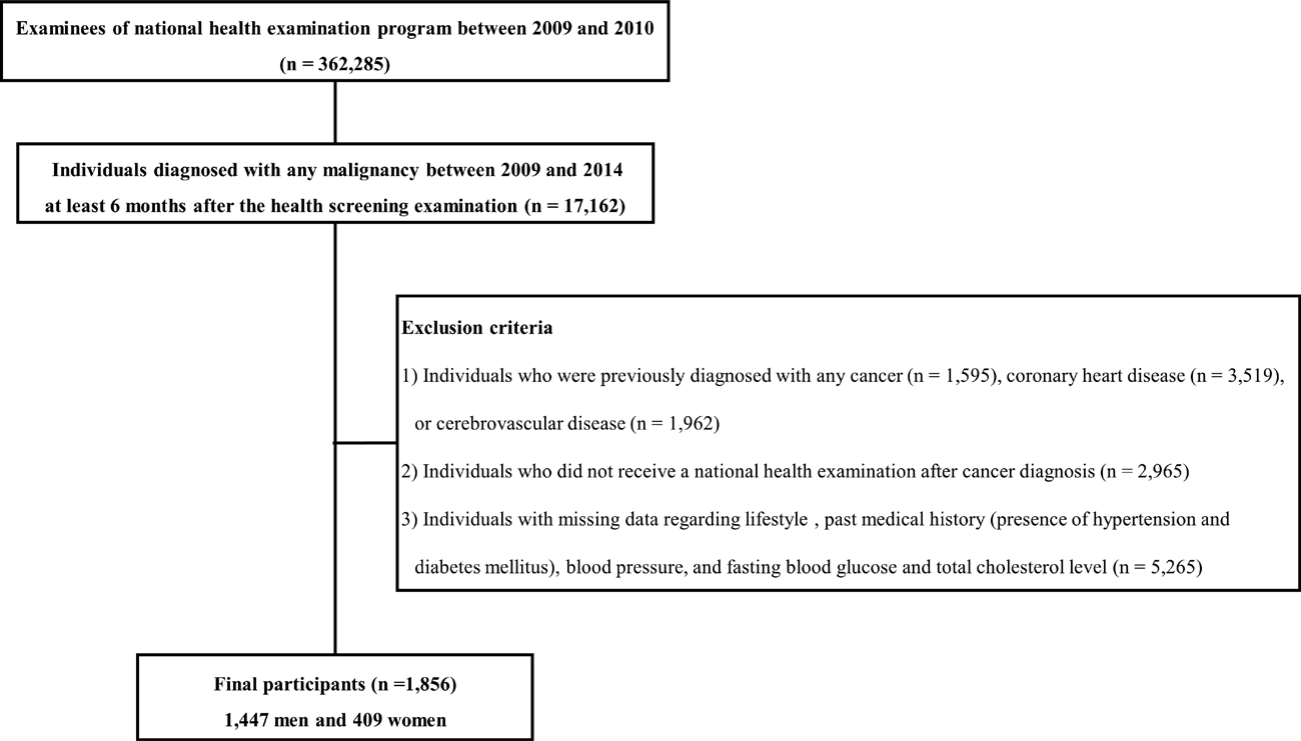
Flowchart of inclusion and exclusion criteria.

Figure 1 illustrates a flow diagram for the inclusions and exclusions in this study. In 2009 and 2010, 362,285 individuals participated in the national health examination programs. Among these, 17,162 individuals diagnosed with any malignancy between 2009 and 2014 at least 6 months after the national health examination were included. Exclusion criteria were: individuals who were previously diagnosed (n = 7076) with any cancer (n = 1595), coronary heart disease (n = 3519), or cerebrovascular disease (n = 1962) based on self-reported questionnaires or diagnostic codes (the International Classification of Diseases [ICD]-10 codes of C00−C99 for cancers, I20−I25 for coronary heart diseases, or I60-69 for cerebrovascular diseases; individuals who did not receive a national health examination after cancer diagnosis (n = 2965); and individuals with missing data regarding lifestyle, past medical history (presence of hypertension and diabetes mellitus), blood pressure, and fasting blood glucose and total cholesterol level laboratory tests (n = 5265). After exclusions, data from 1856 individuals were included in the final analysis.

The Institutional Review Board of Chungbuk National University Hospital approved this study (IRB No. CBNUH 2022-10-012). All authors followed the 1964 Helsinki Declaration. We followed the Strengthening the Reporting of Observational Studies in Epidemiology reporting guideline.

### 2.2. Definitions of weight change, lifestyle, death, study duration, and confounding factors

BMI and body weight change were calculated


$$\begin{array}{l} BMI=\frac{body\;weight\;\left(unit:kg\right)}{height\;{\left(unit:m\right)}^{2}} \end{array}$$

$$\begin{array}{llll} & Body\;weight\;change\left(\%\right)=\; & \frac{\begin{array}{l} body\;weight\;at\;the\;first\;examination\;after\;cancer\;diagnosis\;-body\;weight\;at\;the\;last\;examination\;before\;cancer\;diagnosis \end{array}}{body\;weight\;at\;the\;last\;examination\;before\;cancer\;diagnosis}\; & \times 100 \end{array}$$

Body weight change was categorized into 3 groups: “1) weight loss, ≤ 95% of the baseline body weight; 2) weight maintenance, 95 to < 105% of the baseline body weight; and 3) weight gain, ≥ 105% of the baseline body weight.” Death was defined based on death certificate data including the date and cause of death. If the main cause of death was listed as malignant neoplasm on the death certificate, we defined the death as a cancer-related mortality. Any death including cancer-related mortality was defined as all-cause mortality.

The study start date was defined as the day between the first and second examinations when the individual was diagnosed with any malignant neoplasm. If the patient died, the end date was the date of death. However, if the person did not die during the study period, the end date was either the date of the last clinic visitation or the last national health examination, whichever came most recent.

Lifestyle information was obtained through self-reported questionnaires. Smoking status was classified into never smokers (those who had never smoked tobacco), former smokers (those who smoked in the past but had ceased smoking), and current smokers (those who currently smoke). Alcohol consumption was categorized into nondrinkers (those who had never consumed alcohol), low-risk drinkers (those who were neither nondrinkers nor high-risk drinkers), and high-risk drinkers (those who consumed more alcohol than 56 g/day or 196 g/week for men and 42 g/day or 98 g/week for women).^[16]^ The standard serving of alcohol in Korea was converted to 8 g.^[16]^ Physical activity was classified into 2 groups. Individuals who were not engaged in moderate to vigorous-intensity physical activities or who walked twice or less a week were classified as the inactive group, and those who walked 3 times or more a week were classified as the active group.^[17,18]^

Confounding factors included age, sex, blood pressure, economic status, area of residence, cancer type and diagnosis year, and Charlson comorbidity index (CCI) at baseline. Economic status was stratified into 3 groups based on monthly household income: low, < 30^th^ percentile; middle, 30^th^ to < 70^th^ percentile; and high, 70^th^ to 100^th^ percentile. Area of residence was divided into metropolitan cities and other areas. Metropolitan cities included Seoul, Busan, Incheon, Daegu, Daejeon, Gwangju, and Sejong. CCI, developed in 1984 and validated to measure mortality within 1 year after hospital discharge, is commonly used in clinical research to control the underlying disease effect.^[19,20]^

### 2.3. Statistical analysis

Continuous variables are presented as mean ± standard deviation (SD), and categorical variables are expressed as the number of subjects (percentage). To compare the mean or percentage for each variable, analysis of variance for continuous variables and chi square test for categorical variables were used. To investigate the association between weight change and mortality in patients with cancer diagnosis, Cox proportional hazards regression models for death according to weight change were conducted after adjusting for age, sex, BMI, systolic blood pressure, cancer type and diagnosis year, residential area, economic status, and CCI. Because both high and low blood pressures are closely associated with cardiovascular diseases and mortality, blood pressure was adjusted to control its effects.^[21,22]^ In order to minimize the effect of baseline body weight, further analyses were performed after stratifying the entire population according to BMI with a cutoff value of 25 kg/m^2^.

All tests were 2-sided and the statistical significance level was defined as <0.05. SAS enterprise guide 7.1 (SAS Inc., Cary, NC) and R studio version 3.3.3 (The R Foundation, Vienna, Austria) were utilized for statistical analyses.

## 3. Results

The mean study duration was 7.3 years. Of a total of 1856 individuals, 1447 men and 409 women were included. Table 1 demonstrates baseline characteristics of subjects before cancer diagnosis categorized by sex. The mean age of men and women was 60.0 and 57.5 years, respectively. The male and female BMI was 24.1 and 24.0 kg/m^2^, respectively. The percentage of individuals with CCI ≥ 3 was 38.4% in men and 39.6% in women.

**Table 1:** Baseline characteristics before cancer diagnosis.

	Total	Men	Women	*P* value
Number	1856	1447	409	
Age, years	59.4 ± 8.0	60.0 ± 8.2	57.5 ± 7.0	<.001
BMI, kg/m^2^	24.1 ± 2.8	24.1 ± 2.7	24.0 ± 3.0	.615
SBP, mm Hg	125.8 ± 14.8	127.0 ± 14.6	121.6 ± 14.8	<.001
Smokers, N (%)†				<.001
Never	783 (42.2)	384 (26.5)	399 (97.6)	
Former	512 (27.6)	509 (35.2)	3 (0.7)	
Current	561 (30.2)	554 (38.3)	7 (1.72)	
Drinking status, N (%)†,*				<.001
None	312 (16.8)	124 (8.6)	188 (46.0)	
Low-risk	842 (45.4)	658 (45.5)	184 (45.0)	
High-risk	702 (37.8)	665 (46.0)	37 (9.0)	
Physical activity, N (%)†,**				.033
Inactive	1288 (69.4)	1022 (70.6)	266 (65.0)	
Active	568 (30.6)	425 (29.4)	143 (35.0)	
Economic status, N (%)†				<.001
Low	330 (17.8)	237 (16.4)	93 (22.7)	
Middle	565 (30.4)	418 (28.9)	147 (35.9)	
High	961 (51.8)	792 (54.7)	169 (41.3)	
Residence area, N (%)†				.011
Metropolitan	846 (45.6)	637 (44.0)	209 (51.1%)	
Others	1010 (54.4)	810 (56.0)	200 (48.9%)	
CCI, N (%)†				.790
0	196 (10.6)	150 (10.4)	46 (11.2)	
1	421 (22.7)	335 (23.2)	86 (21.0)	
2	521 (28.1)	406 (28.1)	115 (28.1)	
3 or more	718 (38.7)	556 (38.4)	162 (39.6)	

BMI = body mass index, CCI = Charlson comorbidity index, SBP = systolic blood pressure.

* High-risk drinkers: individuals who drank alcohol 56 g/day or more or 196 g/week or more in men and 42 g/day or more or 98 g/week or more in women.

** Active physical activity: individuals who engaged in the physical activity at least 3 times per week.

† These categorical variables are presented as the number of subjects (percentage).

Table 2 presents the baseline characteristics before cancer diagnosis according to weight change. After cancer diagnosis, 366 patients (19.7%) experienced weight loss >5%, 219 patients (11.8%) gained more than 5% of their body weight, and 1271 patients (64.5%) maintained their body weight. Males represented 77.5% of patients in the weight loss group, 78.0% of patients in the weight maintenance group, and 78.1% of patients in the weight gain group (*P* value = .975). The mean age for the weight loss group was 61.1 years, 59.1 years for the weight maintenance group, and 58.6 years for the weight gain group (*P* value < .001). Baseline BMI was highest in the weight loss group and lowest in the weight gain group (*P* value < .001). Current smokers and individuals residing in metropolitan cities were highest in the weight gain group, while high economic status was highest in the weight maintenance group. Physical activity, residential area, and CCI were not significantly different among the 3 groups (*P* values > .05).

**Table 2:** Baseline characteristics before cancer diagnosis according to weight change.

	Weight loss	Maintenance	Weight gain	*P* value
Number	366 (19.7)	1271 (64.5)	219 (11.8)	
Male, N (%)†	284 (77.5)	992 (78.0)	171 (78.1)	.975
Age, years	61.1 ± 8.5	59.1 ± 7.9	58.6 ± 7.4	<.001
BMI, kg/m^2^	24.7 ± 3.1	24.2 ± 2.5	22.6 ± 3.0	<.001
SBP, mm Hg	127.5 ± 15.2	125.7 ± 14.5	123.9 ± 15.5	.013
Smokers, N (%)†				.023
Never	151 (41.3)	551 (43.4)	81 (37.0)	
Former	108 (29.5)	354 (27.9)	50 (22.8)	
Current	107 (29.2)	366 (28.8)	88 (40.2)	
Drinking status, N (%)†,*				.121
None	72 (19.7)	198 (15.6)	42 (19.2)	
Low-risk	162 (44.3)	573 (45.1)	107 (48.9)	
High-risk	132 (36.1)	500 (39.3)	70 (32.0)	
Physical activity, N (%)†,**				.318
Inactive	249 (68.0)	895 (70.4)	144 (65.8)	
Active	117 (32.0)	376 (29.6)	75 (34.2)	
Economic status, N (%)†				.017
Low	63 (17.2)	228 (17.9)	39 (17.8)	
Middle	109 (29.8)	368 (29.0)	88 (40.2)	
High	194 (53.0)	675 (53.1)	92 (42.0)	
Residence area, N (%)†				.623
Metropolitan	159 (43.4)	583 (45.9)	104 (47.5)	
Others	207 (56.6)	688 (54.1)	115 (52.5)	
CCI, N (%)†				.619
0	38 (10.4)	127 (10.0)	31 (14.2)	
1	86 (23.5)	283 (22.3)	52 (23.7)	
2	102 (27.9)	360 (28.3)	59 (26.9)	
3 or more	140 (38.3)	501 (39.4)	77 (35.2)	

BMI = body mass index, CCI = Charlson comorbidity index, SBP = systolic blood pressure.

* High-risk drinkers: individuals who drank alcohol 56 g/day or more or 196 g/week or more in men and 42 g/day or more or 98 g/week or more in women.

** Active physical activity: individuals who were engaged in the physical activity at least 3 times per week.

† These categorical variables are presented as the number of subjects (percentage).

Table 3 indicates the association between weight change and mortality due to all causes or cancer. Compared to the weight loss group, the weight maintenance group was significantly associated with a lower risk for all-cause and cancer-related mortality, and the weight gain group was significantly associated with only all-cause mortality before adjusting for confounding factors (Model 1). These trends were maintained even after age, sex, BMI, systolic blood pressure, cancer type, cancer diagnosis year, residential area, economic status, and CCI were adjusted for. The hazards ratios (HRs) (95% confidence intervals [CIs]) for weight maintenance were 0.327 (0.189−0.568) for all-cause mortality and 0.431 (0.215−0.867) for cancer-related mortality after full adjustment for confounding factors (Model 3). Fully-adjusted HRs (95% CIs) for the weight gain group were 0.149 (0.044−0.505) for all-cause mortality and 0.289 (0.080−1.045) for cancer-related mortality.

**Table 3:** Association between weight changes before and after cancer diagnosis and all-cause or cancer-related mortality.

		Model 1	Model 2	Model 3
All-cause mortality	Weight loss	Reference	Reference	Reference
	Weight maintenance	0.331 (0.197−0.558)	0.376 (0.222−0.637)	0.327 (0.189−0.568)
	Weight gain	0.184 (0.056−0.609)	0.193 (0.057−0.649)	0.149 (0.044−0.505)
Cancer-related mortality	Weight loss	Reference	Reference	Reference
	Weight maintenance	0.456 (0.235−0.886)	0.502 (0.257−0.982)	0.431 (0.215−0.867)
	Weight gain	0.342 (0.098−1.189)	0.368 (0.103−1.309)	0.289 (0.080−1.045)

Model 1: Not adjusted.

Model 2: Adjusted for age, sex, body mass index, and systolic blood pressure.

Model 3: Adjusted for cancer type, cancer diagnosis year, residential area, economic status, and Charlson comorbidity index, in addition to variables of Model 2.

Table 4 demonstrates these associations after stratifying the entire population according to baseline BMI. Among individuals with BMI < 25 kg/m^2^, weight maintenance and gain were negatively associated with all-cause mortality (HRs [95% CIs], 0.286 [0.138−0.592] in the weight maintenance group and 0.119 [0.027−0.533] in the weight gain group) compared to the weight loss group after fully adjusting for all confounding factors. However, cancer-related mortality was not statistically significant in both weight maintenance and gain groups. Among individuals with BMI ≥ 25 kg/m^2^, the adjusted HR (95% CIs) for all-cause mortality was significant with weight maintenance (0.306 [0.123−0.762]), and that of weight gain was not significant. In addition, adjusted HRs for cancer-related mortality were not significant in either group.

**Table 4:** Association between weight change before and after cancer diagnosis and all-cause or cancer related mortality according to baseline body mass index.

BMI < 25 kg/m^2^		Model 1	Model 2	Model 3
All-cause mortality	Weight loss	Reference	Reference	Reference
	Weight maintenance	0.294 (0.152−0.567)	0.355 (0.181−0.696)	0.286 (0.138−0.592)
	Weight gain	0.131 (0.030−0.568)	0.161 (0.037−0.707)	0.119 (0.027−0.533)
Cancer-related mortality	Weight loss	Reference	Reference	Reference
	Weight maintenance	0.409 (0.172−0.975)	0.457 (0.189−1.102)	0.406 (0.156−1.059)
	Weight gain	0.259 (0.055−1.221)	0.310 (0.065−1.475)	0.253 (0.052−1.239)
BMI ≥ 25 kg/m^2^		Model 1	Model 2	Model 3
All-cause mortality	Weight loss	Reference	Reference	Reference
	Weight maintenance	0.371 (0.157−0.873)	0.378 (0.159−0.901)	0.306 (0.123−0.762)
	Weight gain	0.323 (0.041−2.525)	0.371 (0.047−2.948)	0.230 (0.027−1.946)
Cancer-related mortality	Weight loss	Reference	Reference	Reference
	Weight maintenance	0.506 (0.18−1.421)	0.513 (0.180−1.461)	0.387 (0.129−1.160)
	Weight gain	0.540 (0.065−4.484)	0.593 (0.070−5.038)	0.341 (0.037−3.110)

BMI = body mass index.

Model 1: Not adjusted.

Model 2: Adjusted for age, sex, body mass index, and systolic blood pressure.

Model 3: Adjusted for cancer type, cancer diagnosis year, residential area, economic status, and Charlson comorbidity index, in addition to variables of Model 2.

## 4. Discussion

These findings demonstrated that weight maintenance and gain may reduce the risk of all-cause mortality in patients who were newly diagnosed with any malignant neoplasm based on the NHIS-HEALS cohort. In addition, weight maintenance was significantly associated with a lower risk of cancer-related mortality.

The Korean National Cancer Center reported that 247,952 cancer cases occurred in 2020.^[21]^ Although the rate of increase in cancer incidence has slowed recently, malignant neoplasms still occur in large numbers and are the number one cause of death in Korea.^[1,23]^ In 2022, there were over 2 million cancer survivors in Korea. Thus, Korean health authorities have made various efforts and policies to reduce cancer incidence and mortality, including providing programs regarding cancer prevention, screening, and survivorship.^[5,6]^ Healthy lifestyles, such as smoking cessation and reducing alcohol consumption and increasing physical activity, are strongly recommended to prevent cancer incidence and improve health outcomes for cancer survivors. Maintaining a healthy body weight is one of the important lifestyle habits.

Obesity is a risk factor of cardiovascular disease and certain types of cancer although being overweight or mildly obese reduces the risk of overall death.^[7]^ In addition to obesity, weight loss or gain is significantly associated with a higher risk of all-cause mortality compared to stable body weight maintenance in the general population.^[8,13]^ Several studies have shown that baseline BMI was significantly related to mortality in cancer survivors.^[24,25]^ However, there is a lack of evidence examining the association between weight change and mortality in Korean patients who were newly diagnosed with malignant neoplasms. This study indicated that weight maintenance reduced all-cause and cancer-related mortality in newly diagnosed cancer patients (HRs [95% CIs]: 0.327 [0.189−0.568] for all-cause mortality and 0.431 [0.215−0.867] for cancer-related mortality). In addition, these findings were consistent in the BMI < 25 kg/m^2^ population after stratifying according to BMI. Weight gain reduced all-cause mortality and was marginally associated with cancer-related mortality in the entire population (HRs [95% CIs]: 0.149 [0.044−0.505] for all-cause mortality and 0.289 [0.080−1.045] for cancer-related mortality). Weight gain decreased the risk for all-cause mortality in the BMI < 25 kg/m^2^ population (0.119 [0.027−0.533]). These findings emphasize the importance of maintaining or gaining body weight after initial diagnosis of cancer.

Obesity is closely related to poor prognosis in multiple cancer types. Obesity induces various metabolic dysfunctions and inflammatory processes that increase insulin, insulin-like growth factor, leptin, and interleukin-6, while decreasing adiponectin. These disarrangements activate intracellular signaling through Janus kinase pathway signal transducers and activators of transcription and mitogen-activated protein kinase and phosphatidylinositol 3-kinase pathways, which are frequently dysregulated in carcinogenesis.^[26]^ In addition, adipose tissue produces aromatase that converts androgen to estrogen, which potentiates estrogen signaling and leads to sex hormone-dependent cancers such as breast and ovarian cancer.^[27,28]^ Thus, intentional weight reduction decreased obesity-related cancer incidence.^[29,30]^ Obesity or weight gain cause poor health outcomes in long-term cancer survivors through the development of second primary cancer, recurrence, and cardiovascular diseases.^[31–33]^ Conversely, underweight or weight loss increases the risk of mortality in the advanced cancer population. Weight loss is one of the most common manifestations of cancer. In particular, cancer cachexia, accompanied by a catabolic state and malnutrition, is a devastating and frequently irreversible condition that affects 50% to 80% of advanced cancer patients.^[34]^ Weight loss, including that associated with cancer cachexia, significantly increases cancer mortality. Therefore, weight loss may affect health outcomes according to cancer stage and patient adiposity in different ways. However, the current study showed that weight maintenance or gain may improve survival outcomes in patients who were newly diagnosed with malignant neoplasms.

There are some limitations that need to be considered when interpreting the results of the study. First, the study population was not stratified by sex, and males accounted for a large proportion of the total study population. Second, due to the small number in the study population, classification by cancer type was not possible. However, these limitations are associated with an advantage of being able to explain how to manage cancer patients in general. To minimize the effects of confounding factors, such as cancer types or diagnosis year, these were adjusted for in the Cox proportional hazards regression models. Despite adopting a conservative definition of incident malignant neoplasms, there was still the possibility of false positives. However, the Korean NHIS strictly monitors severe diseases such as cancers, so the number of false positives is likely to be small. The study was unable to consider the stage or pathology type of cancer at diagnosis, which can have a significant impact on mortality. Additionally, information on cancer management strategies, such as surgical removal, chemotherapy, or radiotherapy, was not controlled for in this study due to unavailability of information in the NHIS-HEALS cohort. Finally, the study was unable to distinguish intentional from unintentional weight change and the tissue source of weight change, adipose or muscle tissue. Additionally, weight loss may be a cause of death. To control reverse causality between weight change and death, further analyses were performed after exclusion of individuals who died within 1 year after the second national health examination. Because the population was too small to discriminate the association, statistical significance was not achieved (Data was not shown). However, individuals who maintained or gained body weight after cancer diagnosis had a lower risk of death than individuals who lost weight, even though the hazard ratios were not significant. We believe that the possibility of reverse causality is low.

Despite the limitations, this study has several strengths. The NHIS-HEALS cohort is representative of the entire Korean population based on clinical measurements. Almost all Koreans are enrolled in the NHIS, which is operated by the Korean government as the only insurance institution. The NHIS biennially provides free national physical examination services for the population over 40 years of age. These health examination programs include anthropometric measurements; lifestyle questionnaires; personal medical histories; laboratory test results for blood glucose, lipid profile, and liver enzymes; and cancer screenings for stomach, colorectum, liver, uterine/cervix, and breast. In addition, since the NHIS-HEALS cohort is linked to the death certificate, the cause and date of death are known. This multifaceted investigation was an accurate analysis. We enrolled newly diagnosed cancer patients with available weight within the last 6 months prior to cancer diagnosis. The weight measured at the health screening, not the weight immediately before cancer diagnosis, reflects the patients’ usual weight because weight loss is a common manifestation in cancer. The mean follow-up duration (7.3 years) was relatively long for examining the association between weight change and mortality. In addition, CCI at baseline was controlled. CCI is one of the important indicators to correlate mortality within 1 year after hospital discharge with disease burden.^[19,20]^

In summary, weight maintenance and gain reduced the all-cause mortality in newly diagnosed cancer patients. In particular, maintaining body weight was significantly associated with a lower risk of cancer-related mortality compared to weight loss after cancer diagnosis.

## Author contributions

**Conceptualization:** Yong-June Kim, Hee-Taik Kang.

**Data curation:** Yoon-Jong Bae.

**Formal analysis:** Yoon-Jong Bae.

**Investigation:** Seung Park, Won Tae Kim, Yonghwan Kim, Hee-Taik Kang.

**Methodology:** Yong-June Kim, Seung Park, Hee-Taik Kang.

**Resources:** Yong-June Kim.

**Supervision:** Yong-June Kim, Hee-Taik Kang.

**Writing – original draft:** Hee-Taik Kang.
